# Supplementary material for: Mitigating COVID-19 outbreaks in workplaces and schools by hybrid telecommuting
Source: PLoS Comput Biol. 2021 Aug 26;17(8):e1009264. doi: 10.1371/journal.pcbi.1009264 (PMC8389398; doi:10.1371/journal.pcbi.1009264)
Supplement: S1 Text — (PDF) [file pcbi.1009264.s001.pdf]

S1 Text. Supporting Information

Color coding of measures studied For added readability, in S5, S6 and S7 Figs, just like in Fig 3, the color of the background encodes the parameter studied: probability of outbreak in blue, duration until outbreak in green, and final total number of people infected when there is an outbreak in red.

Error bars. The error bars represent the 95% confidence interval. More specifically, most of our measures are averages of values, with one value for each run of the simulation (e.g. the probability of outbreak is an average of 0-1 values). The standard deviation of the estimate of the mean is approximately equal to the (estimated) standard deviation of the values, divided by the square root of the number of samples. Error bars are set to be 5 times this standard deviation. Because of Chebyshev's inequality, this corresponds to a $1 - 1/5^2 = 96\%$ confidence interval.

Details about the networks

See S1, S2, S3 and S4 Figs.

Results for all contact networks

See S5, S6, S7 and S8 Figs.

Technical details about the simulation

Rounding reals to integers while preserving the mean of the distribution Since the process is discrete, with one discrete step equal to one day, time steps should be defined as integers. To round real random variables drawn from Gamma distributions into integers without changing the mean of the distribution, we used randomized rounding. For example, if $X = 5.4$ then with probability 40% it is rounded to 6 and with the complementary probability 60% it is rounded to 5, ensuring the average rounded value equals 5.4.

Number of executions performed The simulations presented here were performed as follows: for each possible index case of the graph nodes, for each possible day when that person gets infected, do 20 random executions. Because the the On-Off and Rotating strategies have a period of 2 weeks, there are 14 possibilities for the starting day. Consequently, for the high school contact graph, all the results are obtained by averaging over $327 \times 14 \times 20 = 91560$ random executions. For the workplace contact graph, the results are obtained by averaging over 60760 simulations, and for primary schools, the results are obtained by averaging over 67760 simulations.

Probabilistic coupling of executions For easier qualitative comparison of simulation results for different strategies, we used a probabilistic coupling of the random executions. In each run, the following values are coupled: patient initially infected, day of the initial infection, apparition of symptoms for each person, length of each exposed and infectious period for each person, super-spreading transmission factor for each day and each person. Moreover, to decide whether a transmission occurs, we compare the transmission probability with a uniformly random value between 0 and 1. For each (directed) edge of the contact graph, a random value is sampled whenever the origin is infectious and the other endpoint is susceptible; we couple the sequence of those random values. With these choices, we couple six executions: one for each strategy and the one when there is no strategy.

Reproducibility of the simulations Our simulation code uses a fixed seed for the random generator so that all simulations can be reproduced. Each simulation run is identified by its index. More precisely, the k-th simulation run (run k for short) concerns person $i = \left\lfloor \frac{k}{14\times 20} \right\rfloor$ (persons are sorted by increasing id) and starts at day $j = k \mod 14$.

Systematic sensitivity analysis of model parameters

Parameters are described in Table 2

Graph of persistent contacts See S9, S10, S11 and S12 Figs.

Quantifying the heterogeneity of transmissions. In the high school contact graph, the number X of persons infected by the index case follows a negative binomial distribution with mean $R_0^{local} = 1.25$ and dispersion $K \sim 0.5$ (See Table 2; those values are obtained by suitable calibration of parameters p_0 and p_{super}). In particular, X equals 0 with probability ~ 0.5, equals 1 with probability ~ 0.2, and equals 2 with probability ~ 0.1. Thus, with the complementary probability 20%, we have $X \geq 3$. Thus, 20% of the situations lead to a fraction of $(1.25 - 0.5 \times 0 - 0.2 \times 1 - 0.1 \times 2)/1.25 = 68\%$ of the infections by the case index. See S13 Fig for a sensitivity analysis w.r.t. p_0 and p_{super}.

Surprisingly, in panel (b) of S13 Fig we observe that, the more dispersion in the shape of super-spreaders, the less the probability of outbreak and the fewer people get infected. This can be explained intuitively by thinking about an extreme case of dispersion. Imagine that with probability 1/1000, there is a super-spreading factor of 1000, and with the complementary probability 999/1000, the super-spreading factor equals 0. Then, for most executions, the index case is not a super-spreader and infects no one. In 0.1% of the cases, the index case is a super-spreader and infects all of its neighbors, however none of them will be a super-spreader and propagate the infection. The event that the index case and at least one of its neighbors are both super-spreaders has probability about equal to the number of neighbors times 0.000001, too small to ever occur in the simulations.

Symptoms See S14 and S15 Figs.

Durations of each period See S16 and S17 Figs.

Selected transmission trees

See S19, S20, S21, S22 Figs.
