# Supplementary figures and images for: Mitigating COVID-19 outbreaks in workplaces and schools by hybrid telecommuting
Source: PLoS Comput Biol. 2021 Aug 26;17(8):e1009264. doi: 10.1371/journal.pcbi.1009264 (PMC8389398; doi:10.1371/journal.pcbi.1009264)

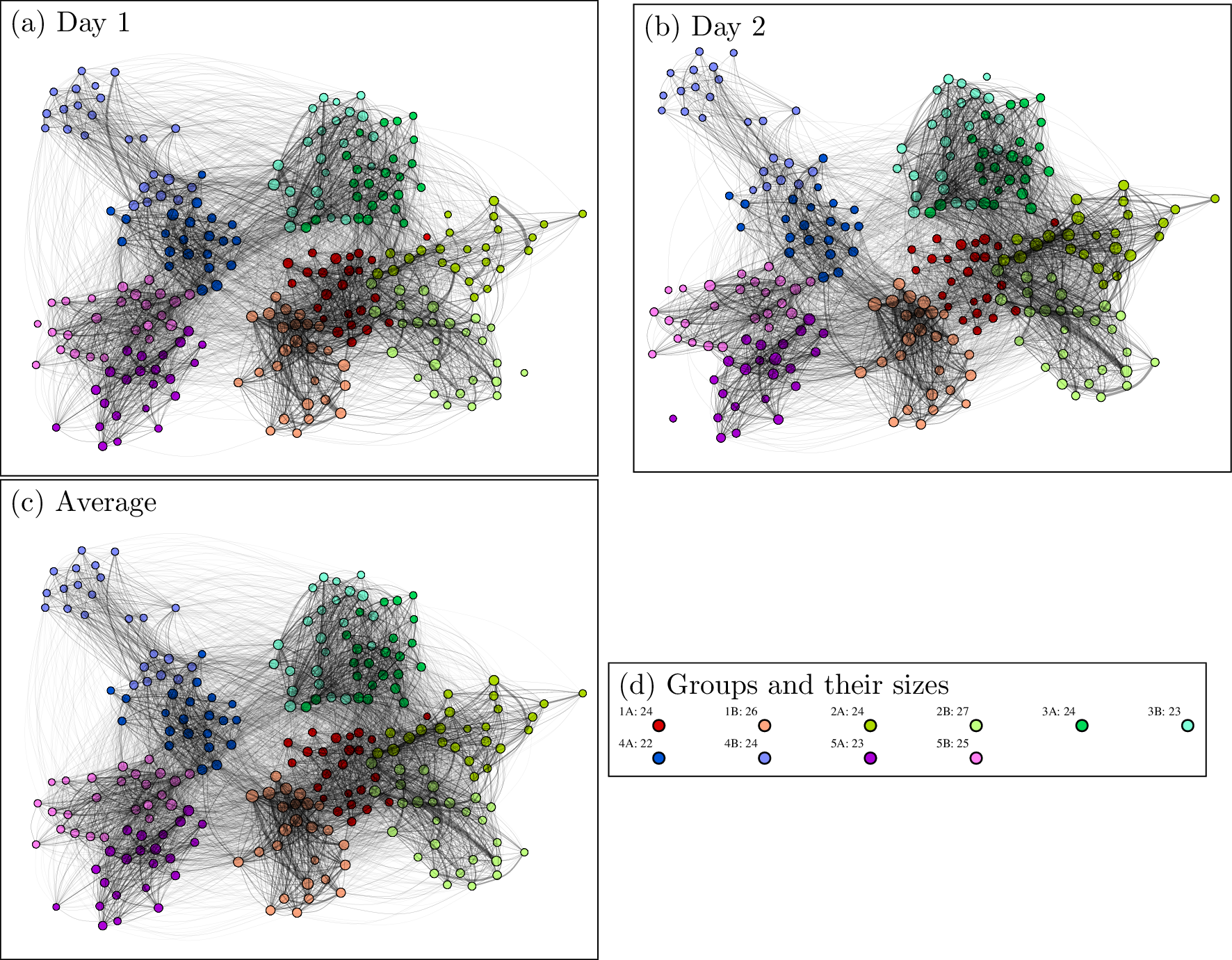

Supplement: S1 Fig — The two days of the trace correspond to Thursday and Friday. Each day is represented by a graph where a node corresponds to an individual, and an edge corresponds one or several face contacts. Edge width corresponds to the number of contacts. Node sizes correspond to weighted degrees. Node colors correspond to known groups which are classes. There size vary between 22 and 27. We observe many contacts between classes of the same grade (e.g. 5A and 5B). (TIF) [file pcbi.1009264.s002.tif]

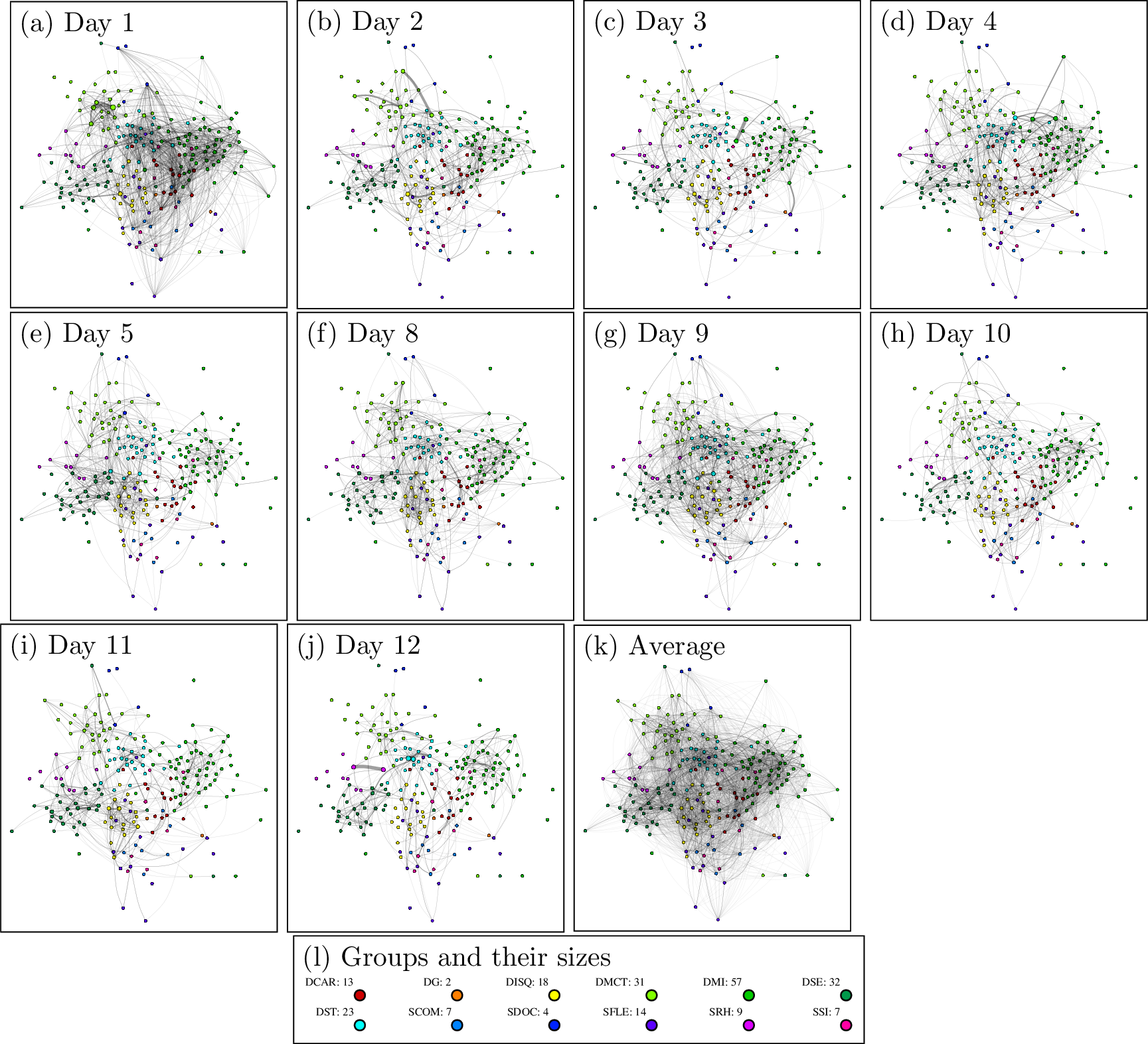

Supplement: S2 Fig — The trace lasts over two weeks and contains contacts only during working days. Each day is represented by a graph where a node corresponds to an individual, and an edge corresponds one or several face contacts. Edge width corresponds to the number of contacts. Node sizes correspond to weighted degrees. Node colors correspond to known groups which are departments. Their size vary from 2 to 57, most of them contain at most 32 persons. (TIF) [file pcbi.1009264.s003.tif]

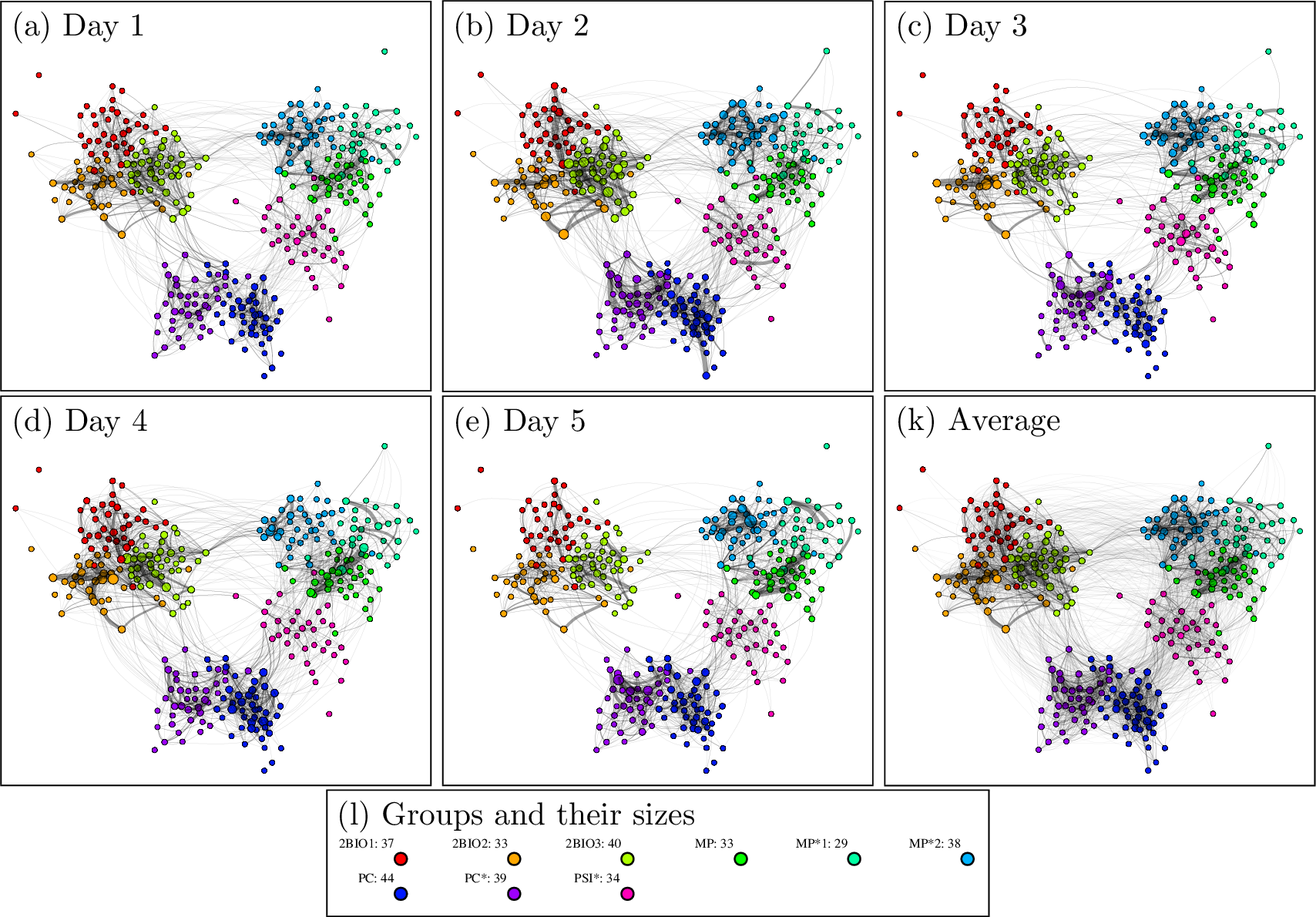

Supplement: S3 Fig — The trace lasts during the 5 working days of a week. Each day is represented by a graph where a node corresponds to an individual, and an edge corresponds one or several face contacts. Edge width corresponds to the number of contacts. Node sizes correspond to weighted degrees. Node colors correspond to known groups which are classes. Their size vary from 29 to 44. (TIF) [file pcbi.1009264.s004.tif]

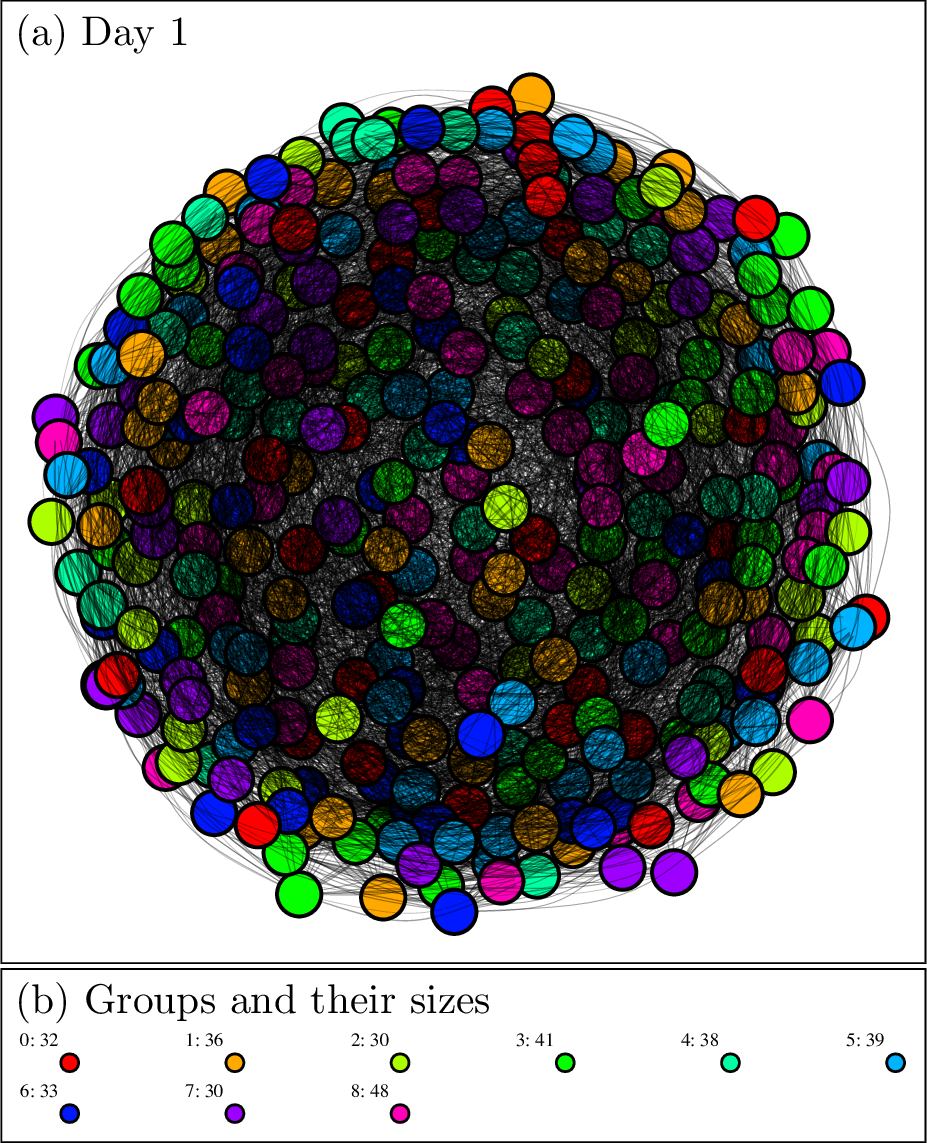

Supplement: S4 Fig — The trace lasts one day (average graph is identical to Day 1). It is represented by a graph where a node corresponds to an individual, and an edge corresponds one or several face contacts. The graph is calibrated so that its main parameters (total number of nodes, of edges, and of contacts) match those of the high-school average graph: more precisely, each edge is generated by selecting uniformly at random two nodes with one associated contact (rejecting loops and already generated pairs) and each of the remaining contacts is associated to an edge selected uniformly at random among the previously generated edges. Edge width corresponds to the number of contacts that were associated to it. Node sizes correspond to weighted degrees. Node colors correspond to groups which were selected uniformly at random for each node within 9 fixed groups. Their size vary from 30 to 48. (TIF) [file pcbi.1009264.s005.tif]

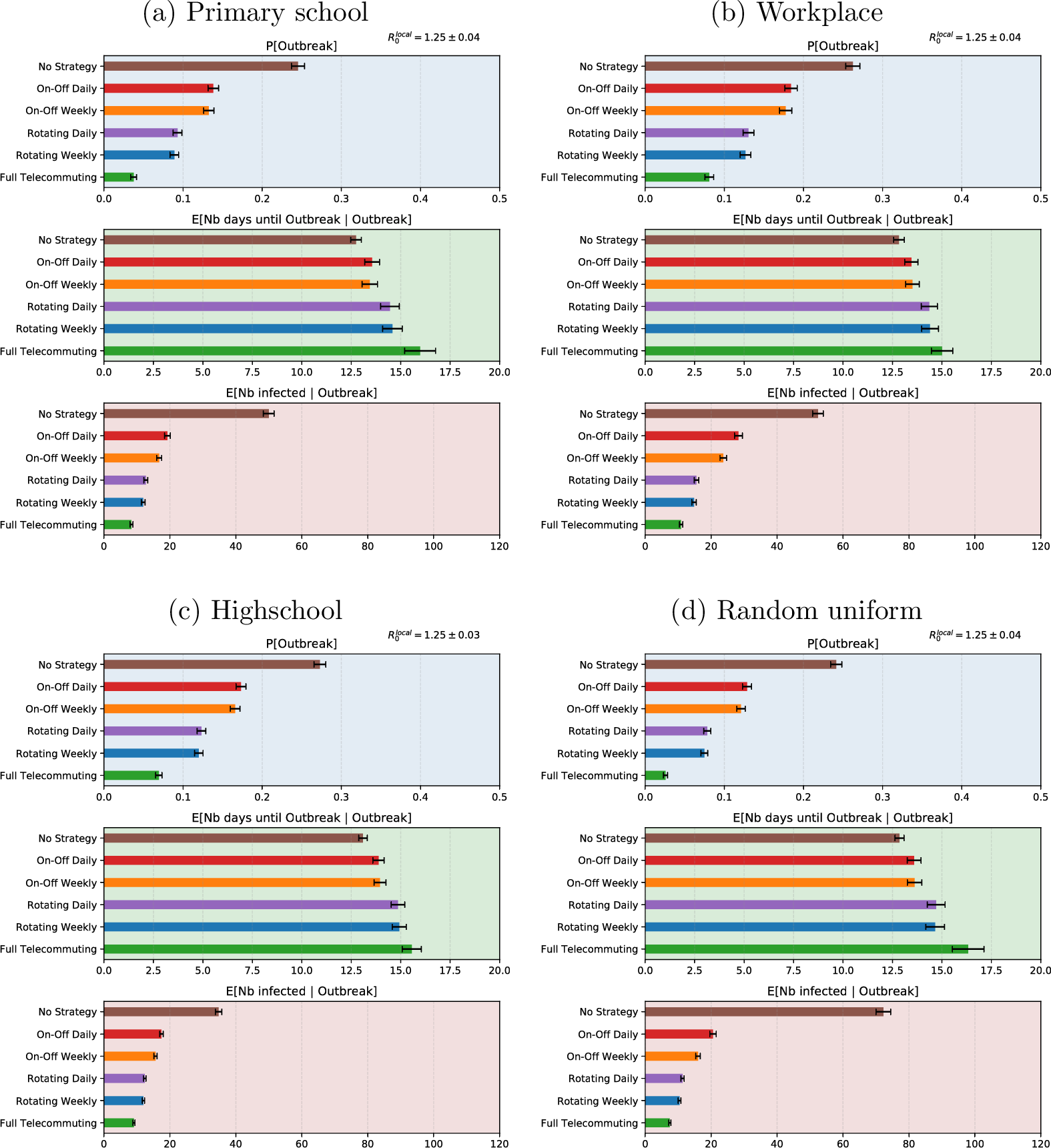

Supplement: S5 Fig — Panel (c) is identical to Fig 3. We see that regardless of the contact graph, the ranking of the strategies by effectiveness is the same, thus the qualitative results are robust. Note that the quantitative results are also similar from graph to graph, with the exception of the total number of infected people: for the high school contact graph (c), it equals 34.8, whereas for the synthetic random graph (d), it equals 72.3. That happens in spite of the fact that the random graph is calibrated to be the same as the high school graph in terms of number of nodes, edges, and contacts: thus, the difference is due to the expansion of the random graph, which contrasts with the high school group structure. (TIF) [file pcbi.1009264.s006.tif]

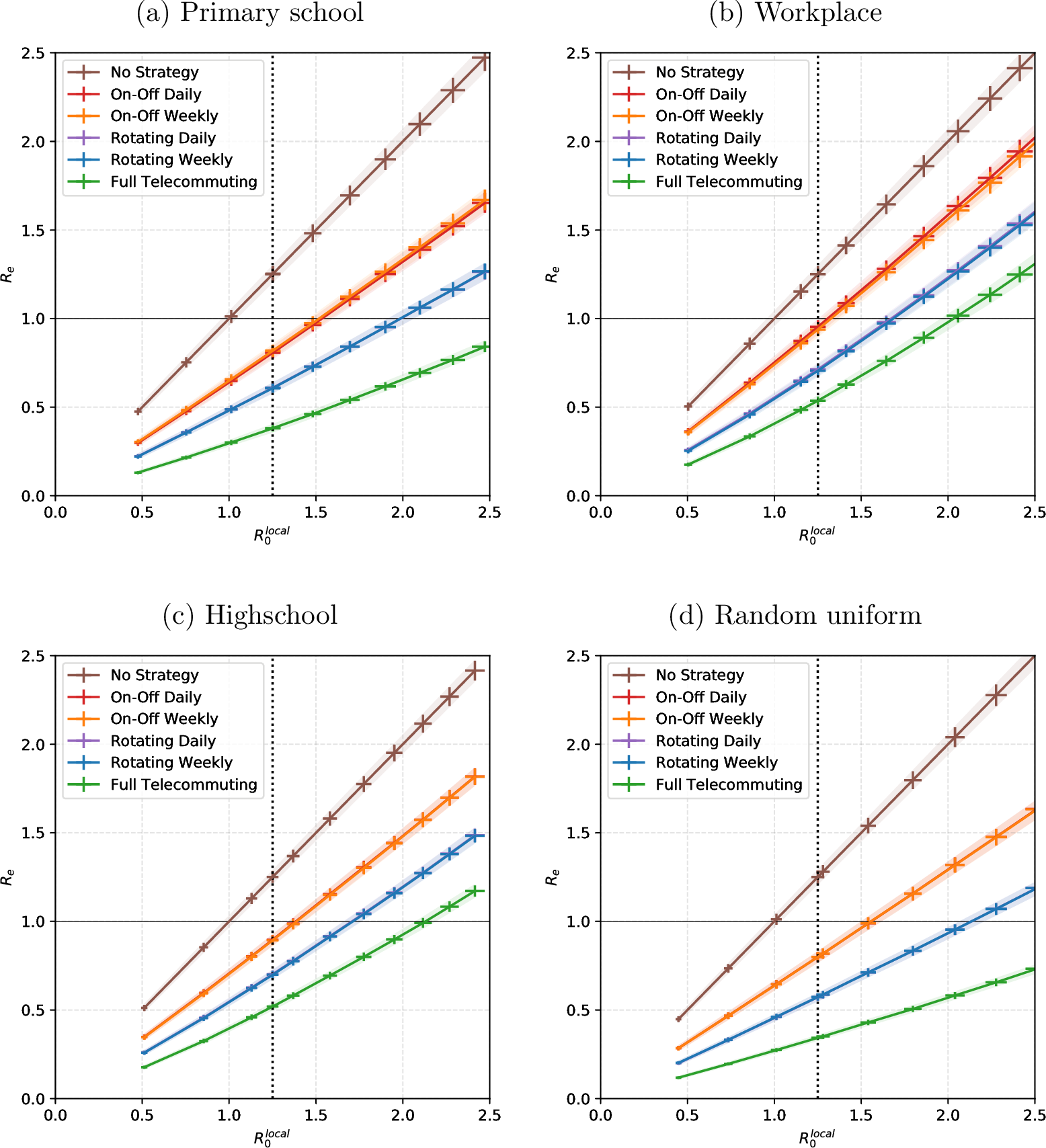

Supplement: S6 Fig — Panel (c) is identical to Fig 4. Qualitatively, we see that the order between the curves is the same for all contact graphs and all values of R0local, so that result is robust. The weekly and daily alternations are indistinguishable for this measure. Quantitatively, if we focus on the largest R0local such that On-Off leads to Re < 1, we see that it depends significantly on the underlying contact graph: R0local=1.52 for primary schools, 1.30 for the workplace, 1.38 for the high school, and 1.55 for the random graph. (TIF) [file pcbi.1009264.s007.tif]

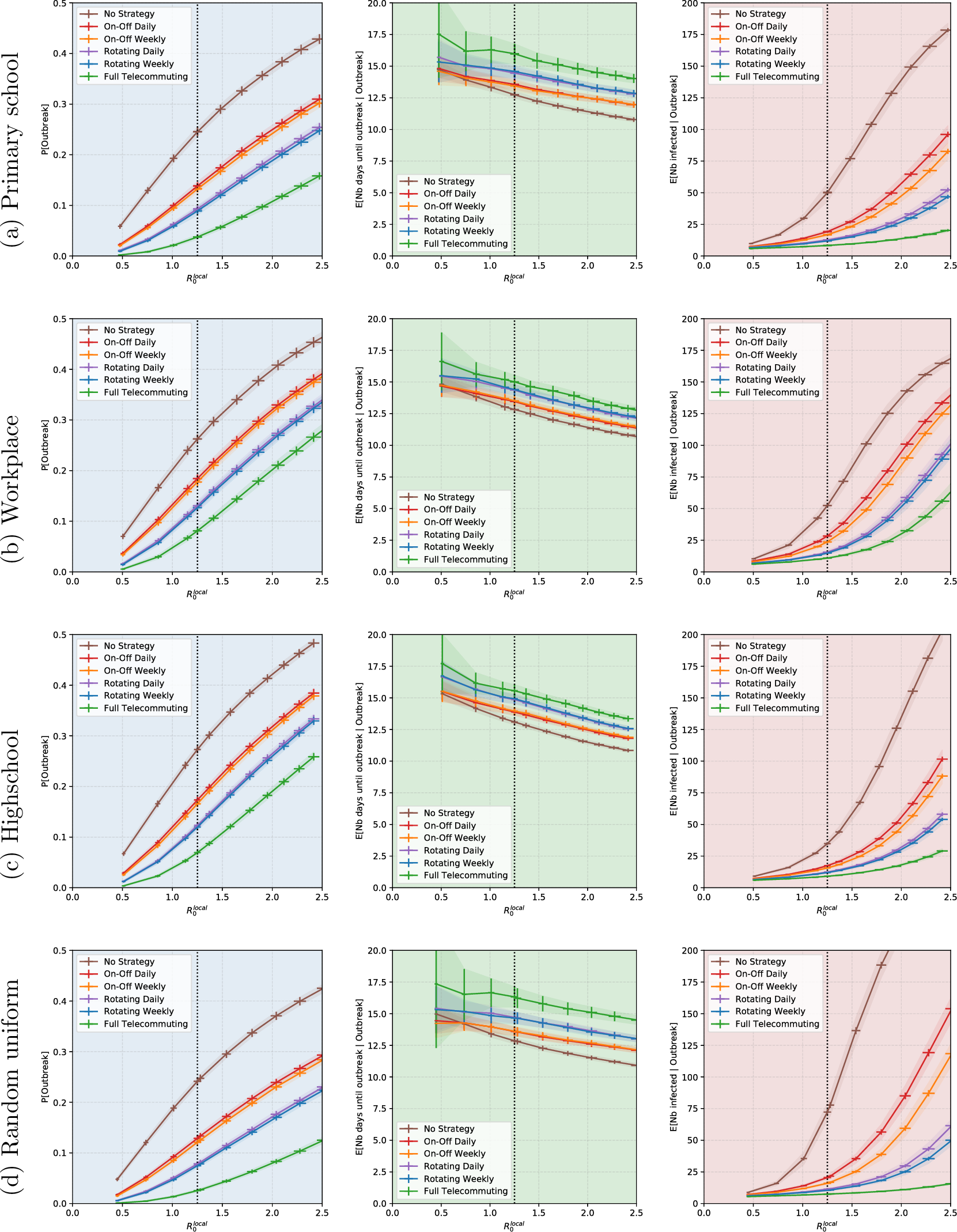

Supplement: S7 Fig — For all contact networks, we performed a sensitivity analysis of the results of S5 Fig w.r.t. R0local (or equivalently, to the parameter p.) We see that the probability of an outbreak is sensitive to the value of R0local: for example, for On-Off, as R0local varies from 1 to 1.5, it goes from 21% to 33%. However, it is not so sensitive to the choice of contact graph: when there is no strategy, for the base case R0local=1.25 it is around .25 for all graphs. The number of days until an outbreak, around two weeks, is fairly robust and shows little sensitivity to either R0local or the choice of contact graph. The final number of people infected conditioned on an outbreak is the most sensitive quantity, both to the value of R0local and to the choice of contact graph. (TIF) [file pcbi.1009264.s008.tif]

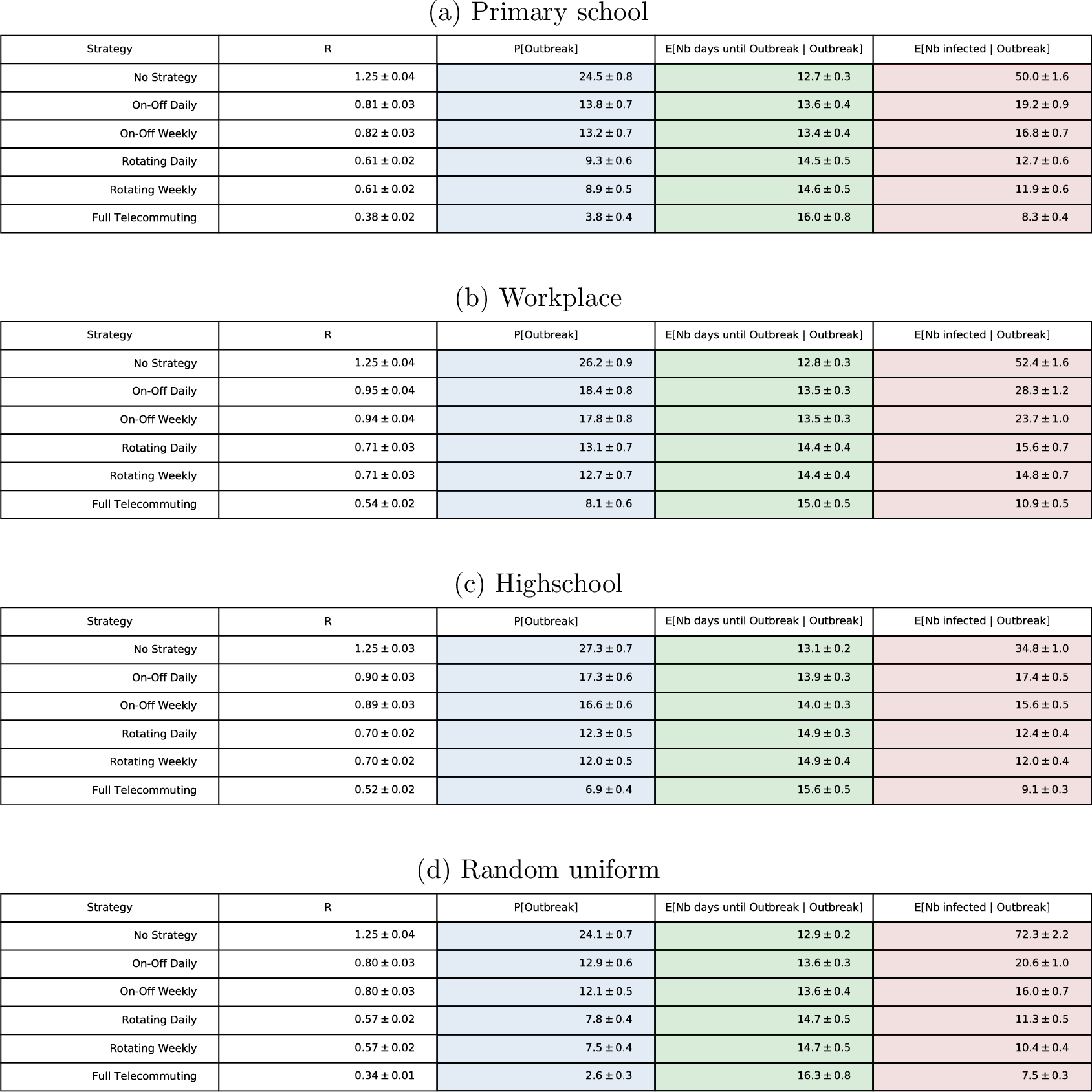

Supplement: S8 Fig — Numerical data of S5–S7 Figs when R0local=1.25. (TIF) [file pcbi.1009264.s009.tif]

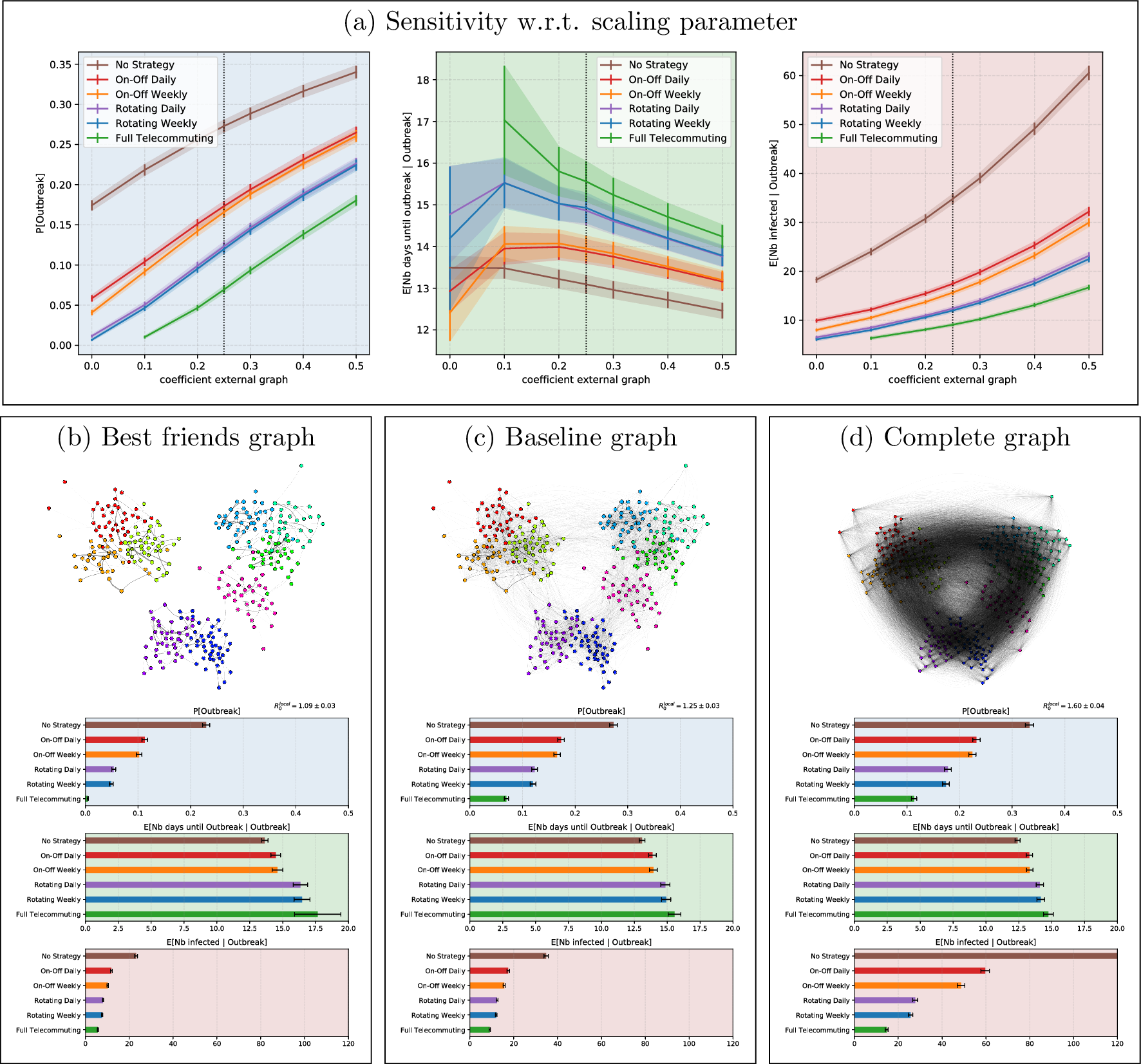

Supplement: S9 Fig — In part (a), we do a sensitivity analysis when we vary the intensity of persistent contacts (baseline: 25%). The baseline case in (a) corresponds to the vertical dotted line, whose intersection with the curves of the strategies gives the values of Fig 3. We see that, the more persistent contacts there are, the worse it is for the epidemic, but that the variation is smooth. Parts (b), (c) and (d) we do a sensitivity analysis in which we vary the structure of the persistent contacts graph, while keeping the total number of contacts unchanged. Part (c) is the baseline case and is an identical copy of Fig 3, for ease of comparison. Part (d) takes a complete homogeneous graph for the persistent contacts graph. Part (b) is a construction of what we call a best friends graph, constructed in the following two steps: First, each person lists their neighbor by order of decreasing number of contacts, stopping as soon as they reach 25% or their total number of contacts. This creates a directed graph in which many arcs carry 0 contacts. Second, we make it symmetric by putting on each edge {u, v} the average of the number of contacts on arc(u, v) and on arc (v, u). We observe that the results are sensitive to the structure. The best friends graph propagates the epidemic the least, the complete graph propagates it the most, and the baseline graph is intermediate. For example, regarding the probability of outbreak, when there is no strategy the probability is 33% for the complete graph, 27% for the baseline graph, and 23% for the best friends graph. Regarding the total number of persons infected when there is an outbreak, when there is no strategy we have 151.8 for the complete graph (the bar actually goes beyond the figure), 34.8 for the baseline graph, and 23.1 for the best friends graph. When the daily On-Off strategy is used, the numbers are 59.7 for the complete graph, 17.4 for the baseline graph, and just 11.8 for the best friends graph. We see that the structure of contacts [file pcbi.1009264.s010.tif]

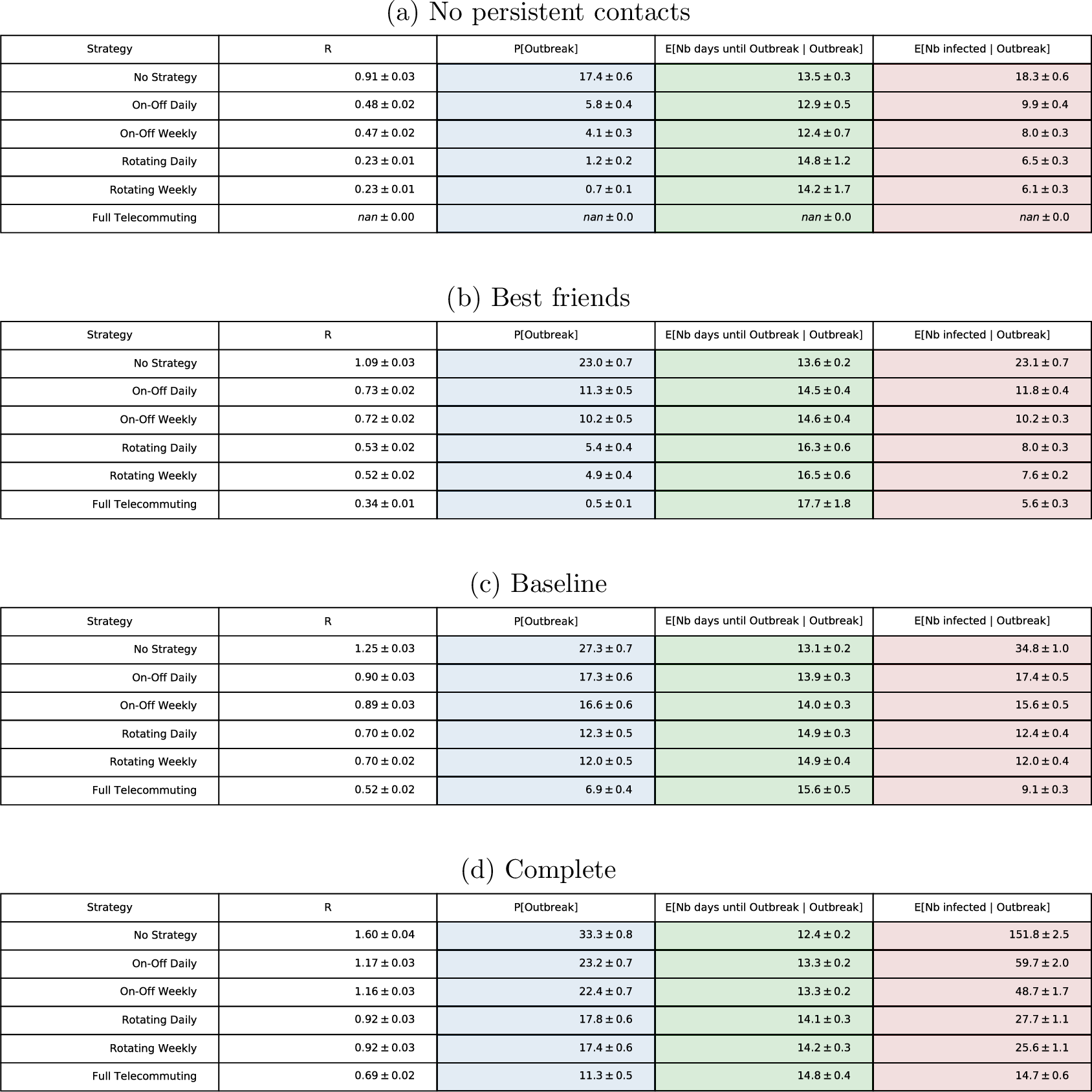

Supplement: S10 Fig — Thus, for the high school contact network, compared to having no strategy, Daily On-Off reduces the reproduction number by 1 − 0.48/0.91 = 47% and Daily Rotation reduces it by 1 − 0.23/0.91 = 75%. The improvement of weekly strategies over their daily analog is less than 2%. (TIF) [file pcbi.1009264.s011.tif]

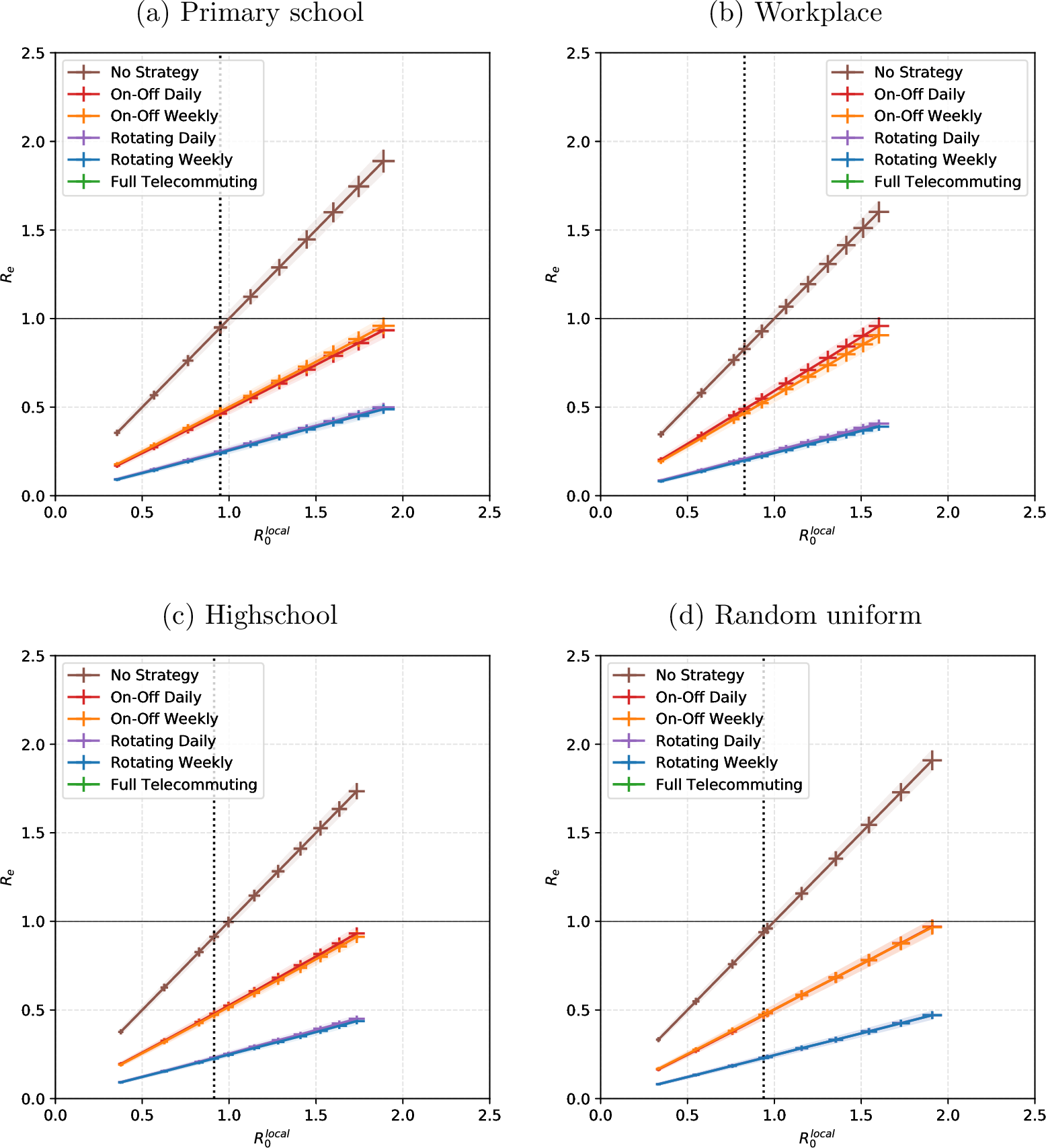

Supplement: S11 Fig — (TIF) [file pcbi.1009264.s012.tif]

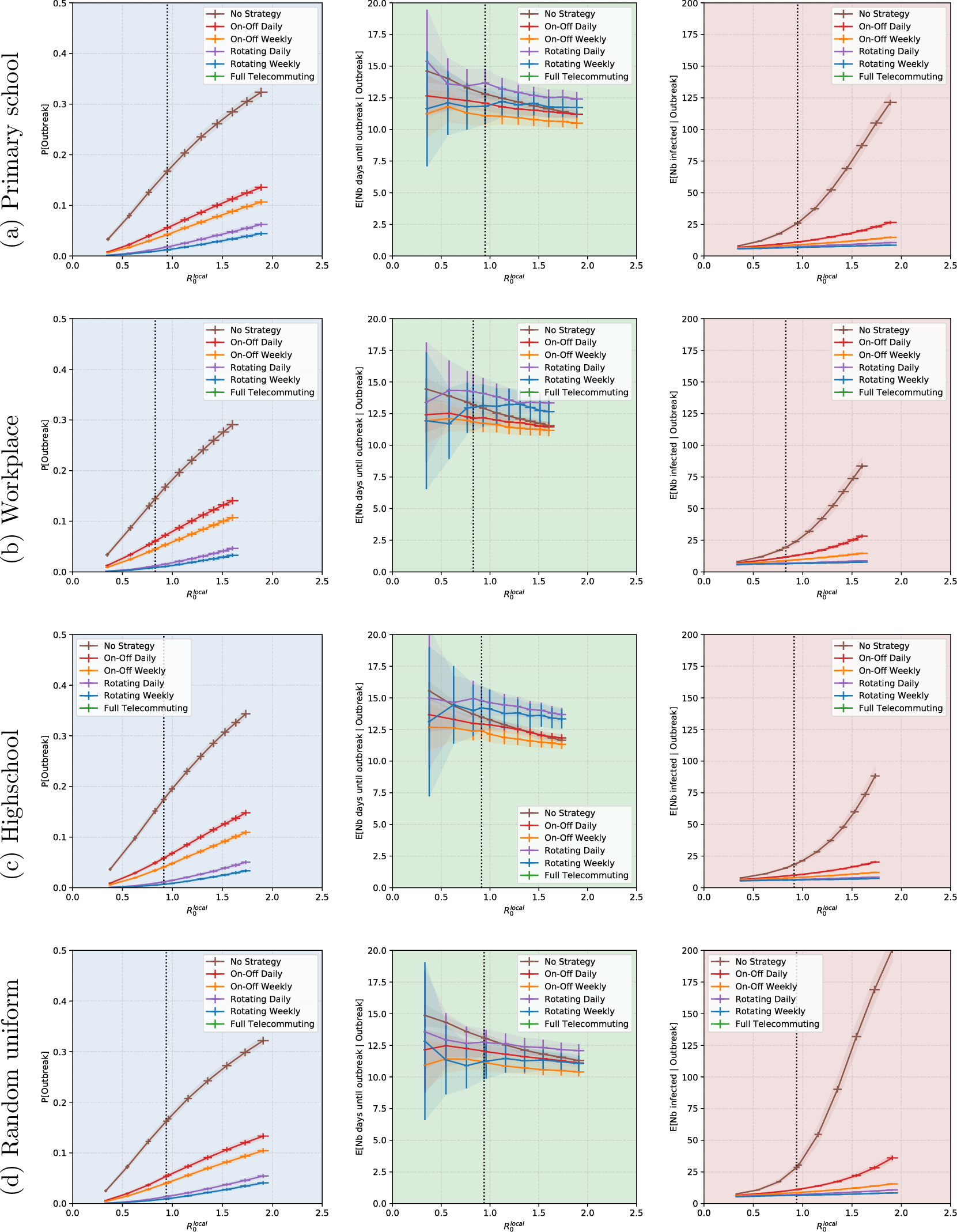

Supplement: S12 Fig — (TIF) [file pcbi.1009264.s013.tif]

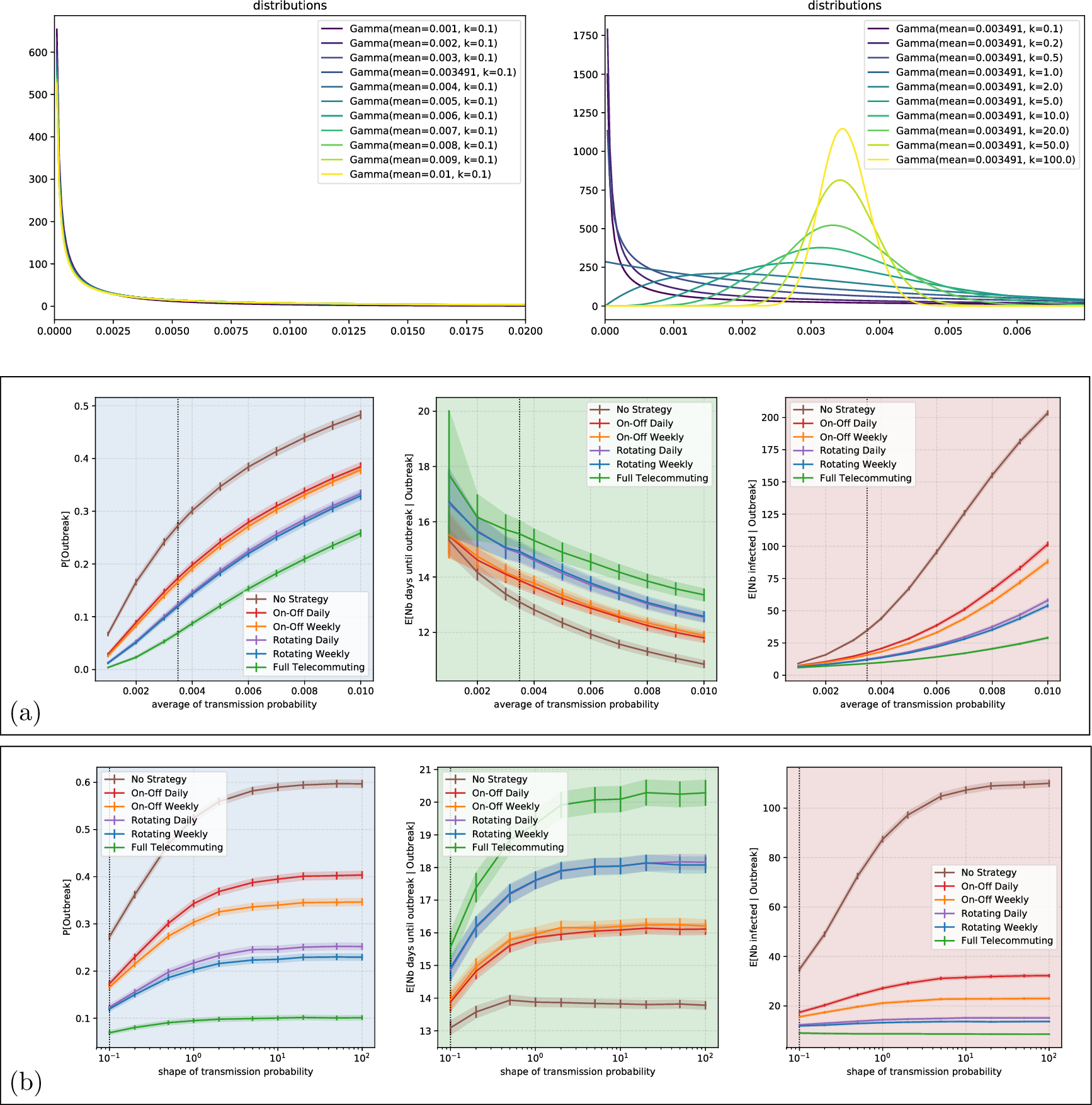

Supplement: S13 Fig — In (a), we look at the parameters as a function of R0local, which varies by changing the value of the probability p of symptomatic transmission (baseline R0local=1.25 corresponding to p = 0.0035, and R0local=1 corresponds to p = 0.025). We do not observe a phase transition in which the number of infected people would explode when R0local becomes greater than 1, but instead, we observe a smooth increase. This is probably due to the small size of the graph (327 nodes), too small to see the theoretical asymptotic behavior as the number of nodes goes to infinity. In (b), we look at the parameters by changing the shape of the super-spreading distribution (gamma of mean 1, baseline shape value 0.1). The baseline case corresponds to the vertical dotted line at R0local=1.25, whose intersection with the curves of the strategies gives the values of Fig 3. (TIF) [file pcbi.1009264.s014.tif]

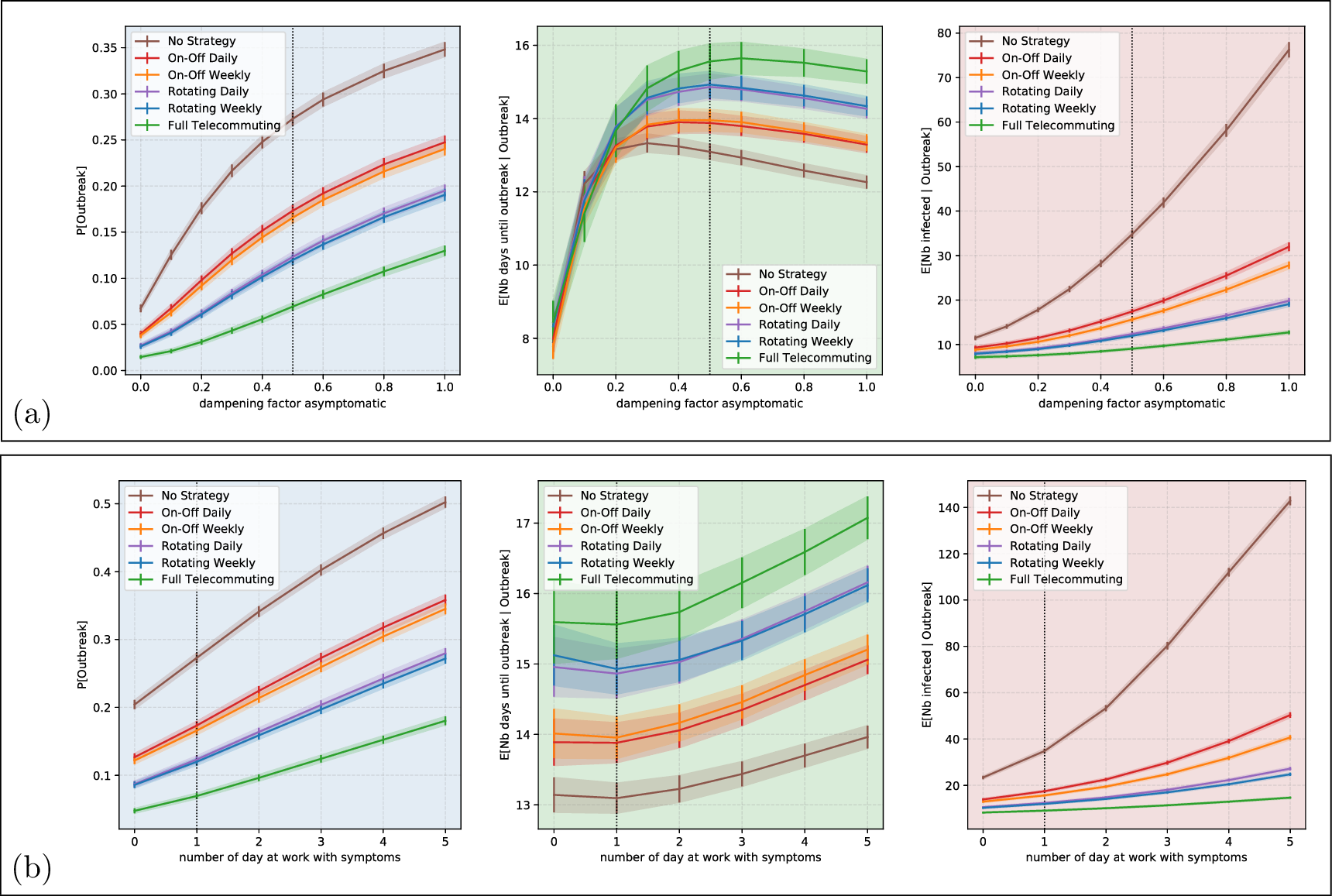

Supplement: S14 Fig — (a) the difference of infectiousness of an asymptomatic person compared to that of a symptomatic person (baseline: 1/2); here there is a tradeoff in the duration until outbreak, conditioning on existence of an outbreak: when asymptomatic persons are almost not infectious, the epidemic evolution is driven by symptomatic persons, who are only able to contaminate others in the first few days before they isolate, so when outbreaks do happen, they happen more quickly; at the other end of the scale, when most asymptomatic people are just as infectious as symptomatic people, they are infectious for many days but because they are more contagious, they infect people earlier on. (b) the number of days during which a symptomatic individuals continues going to school or work after developing symptoms (baseline: 1 day). The baseline case corresponds to the vertical dotted line, whose intersection with the curves of the strategies gives the values of Fig 3. Part (b) suggests that changing behavior so that a person self-isolates as soon as she develops symptoms is very effective to reduce the dissemination of the epidemic in her contact network. (TIF) [file pcbi.1009264.s015.tif]

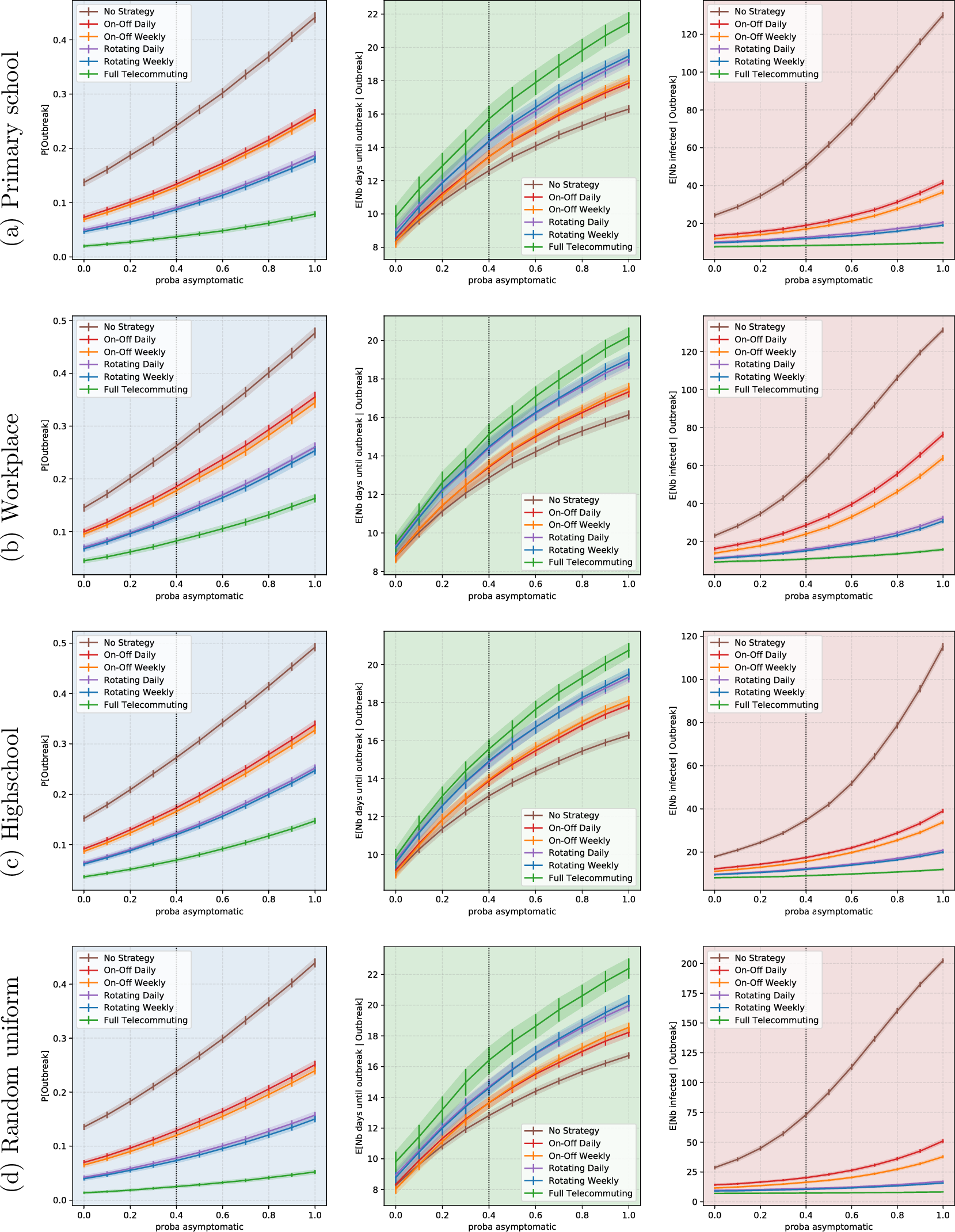

Supplement: S15 Fig — (TIF) [file pcbi.1009264.s016.tif]

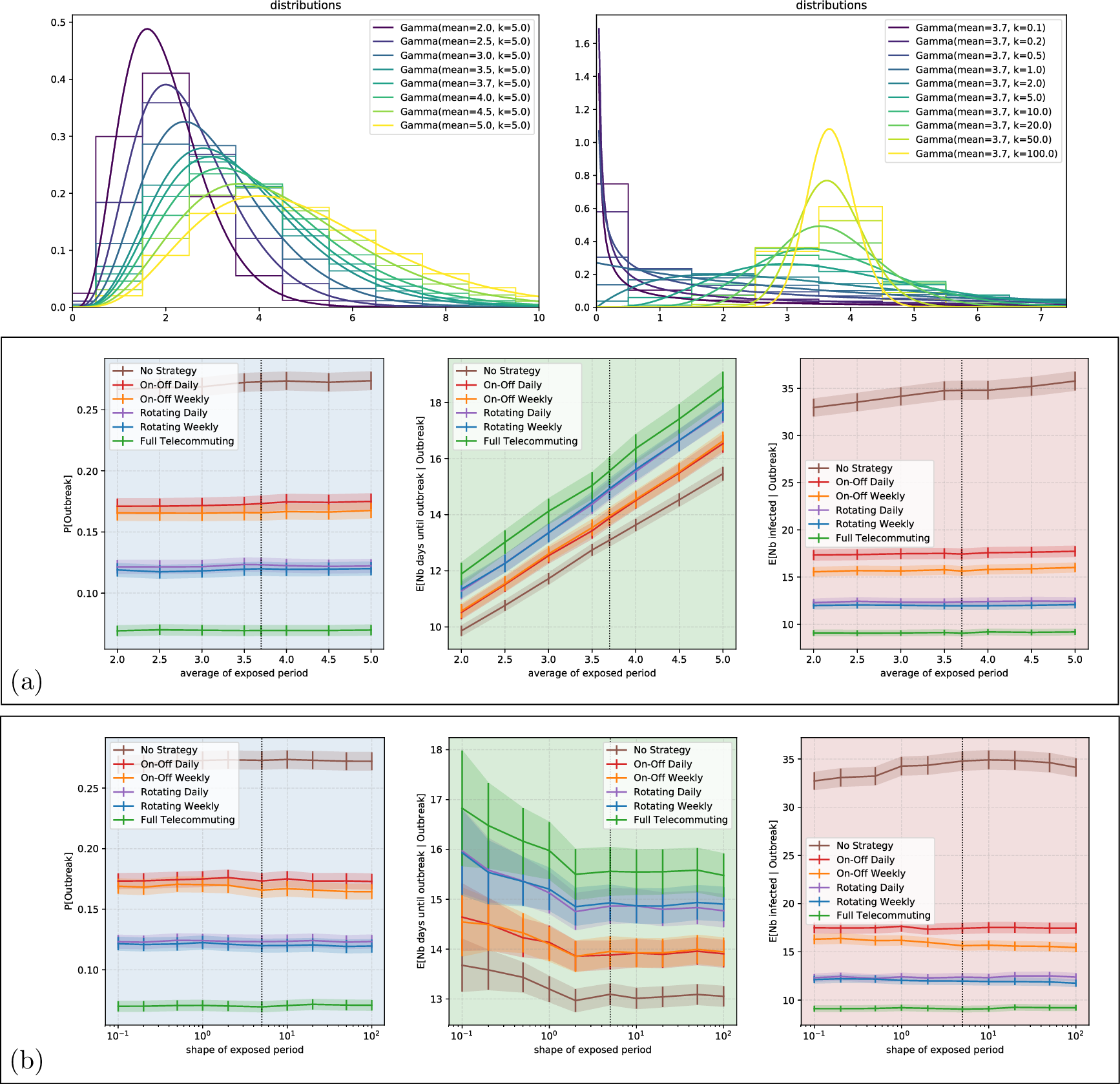

Supplement: S16 Fig — (a) the mean length of the exposed period (baseline: 3.7 days); (b) the shape of the distribution of the exposed period (baseline: 5). The baseline case corresponds to the vertical dotted line, whose intersection with the curves of the strategies gives the values of Fig 3. Unsurprisingly, the longer the exposed period, the more time it takes before 5 people are infected; otherwise the distribution of the exposed period has little impact on the results. (TIF) [file pcbi.1009264.s017.tif]

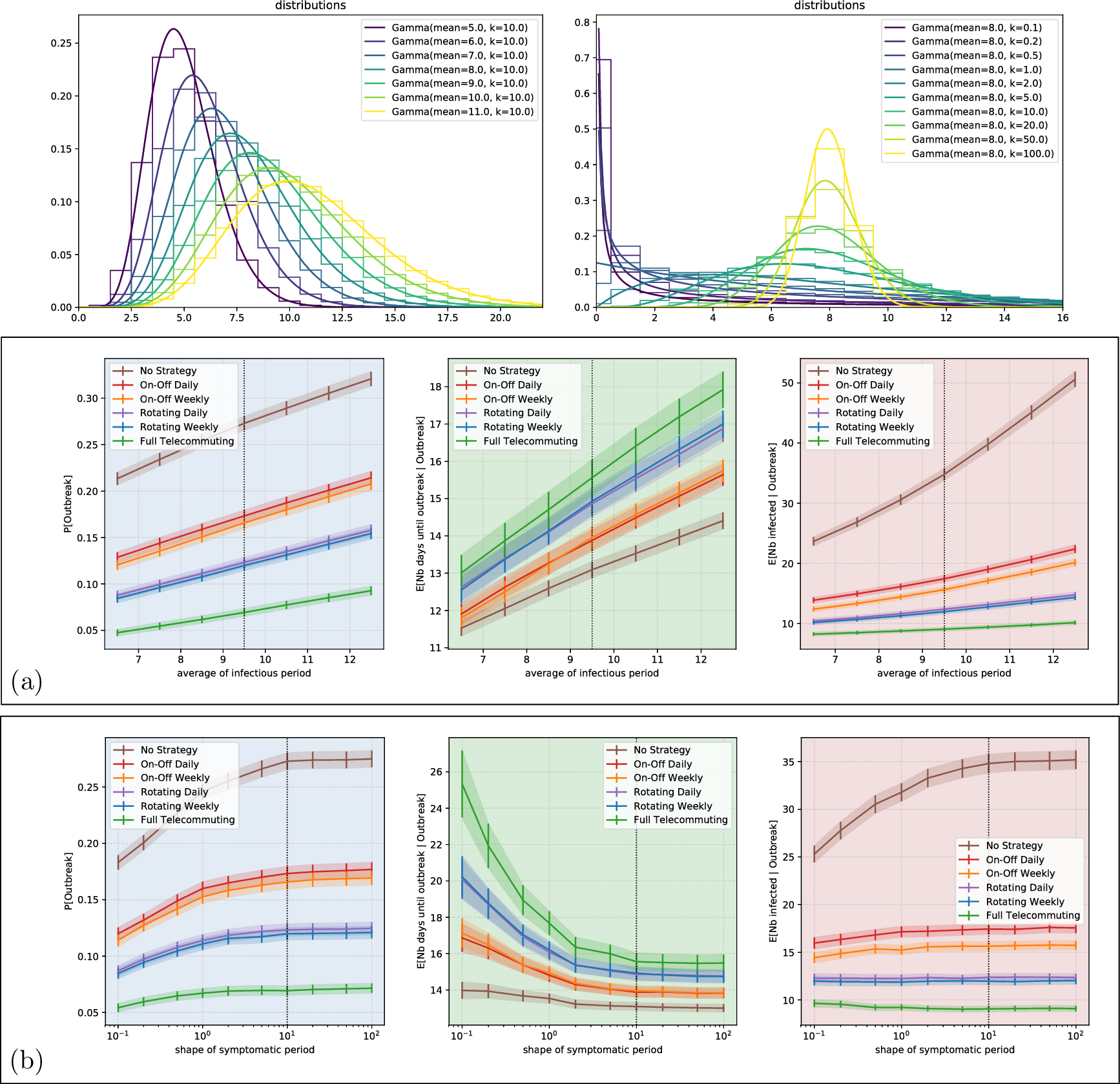

Supplement: S17 Fig — (a) the mean length of the infectious period (baseline: 9.5 days); (b) the shape of the distribution of the remaining of the infectious period after the first 1.5 days (baseline: 10). The baseline case corresponds to the vertical dotted line, whose intersection with the curves of the strategies gives the values of Fig 3. The variations are monotone and smooth. (TIF) [file pcbi.1009264.s018.tif]

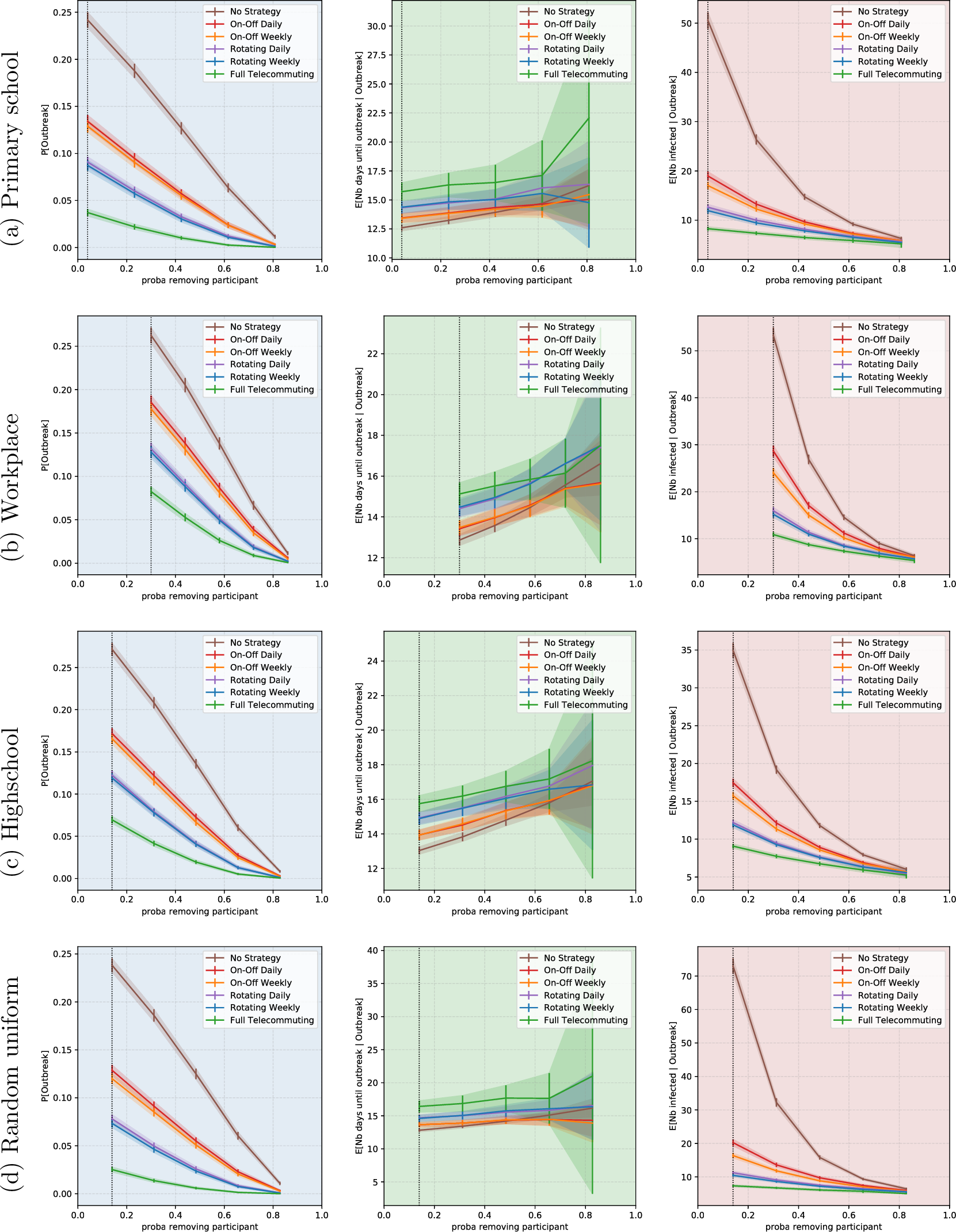

Supplement: S18 Fig — The primary school contact network is missing 4% of the children, who did not participate in the Sociopatterns study. Our baseline results for primary schools are therefore on the 4% vertical line. To analyze the effect of removing participants, we remove additional participants uniformly at random, starting from the data with 4% missing and going all the way to 50%. We proceed similarly for high schools (baseline: 14%) and for the workplace (baseline: 30%). We see that, unsurprisingly, removing people reduces the probability of outbreak, reduces the expected final number of people infected conditioned on an outbreak, and increases the number of days until there is an outbreak. Thus, our quantitative results seem to be an underestimate of the situation in the actual contact network. Note that the value of p was not recalibrated for each new network, hence sparser networks have a lower R value. (TIF) [file pcbi.1009264.s019.tif]

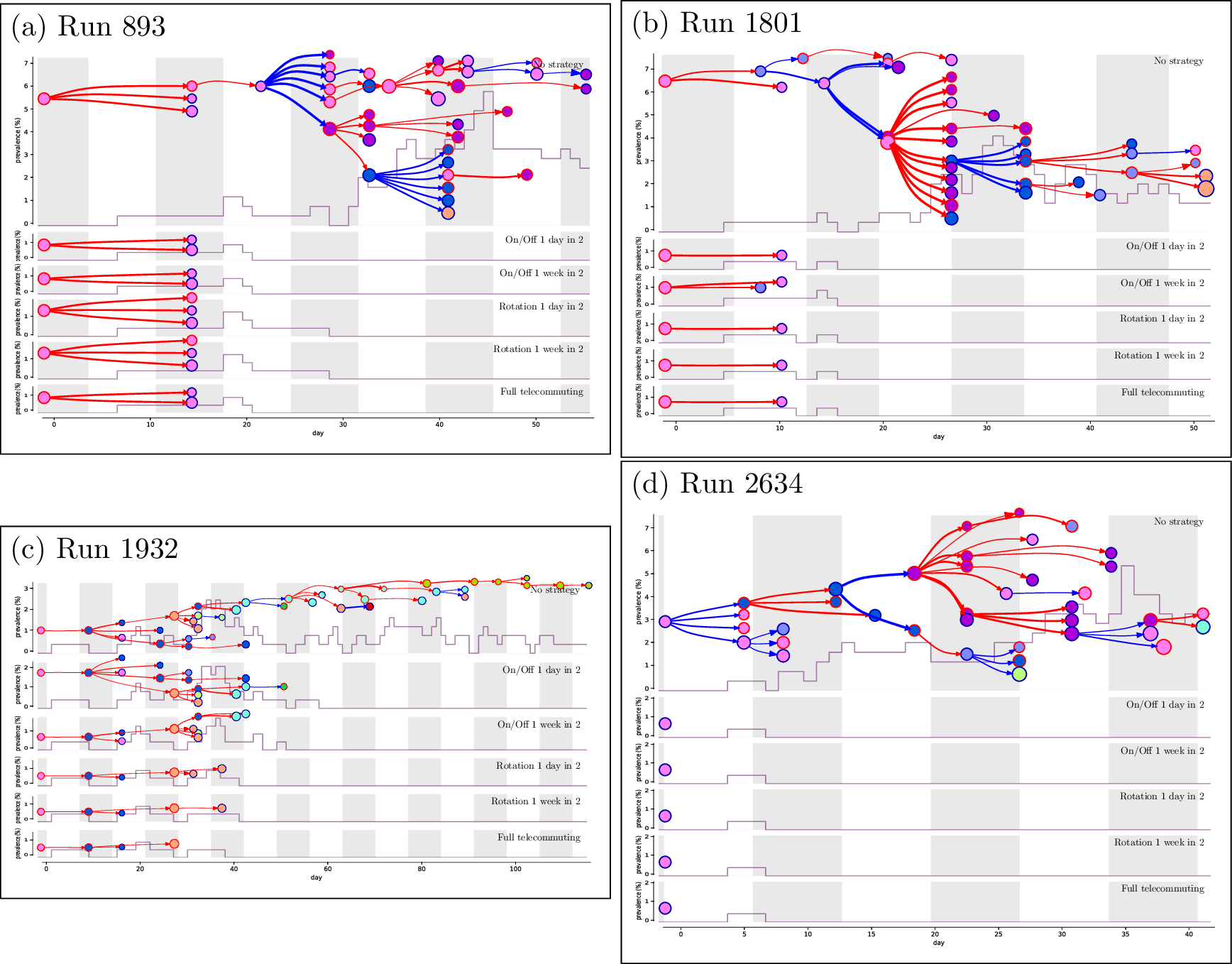

Supplement: S19 Fig — Among the runs producing an outbreak under no strategy, we selected the first four that produce a median number of infections when we do not implement a strategy, that is 37. (TIF) [file pcbi.1009264.s020.tif]

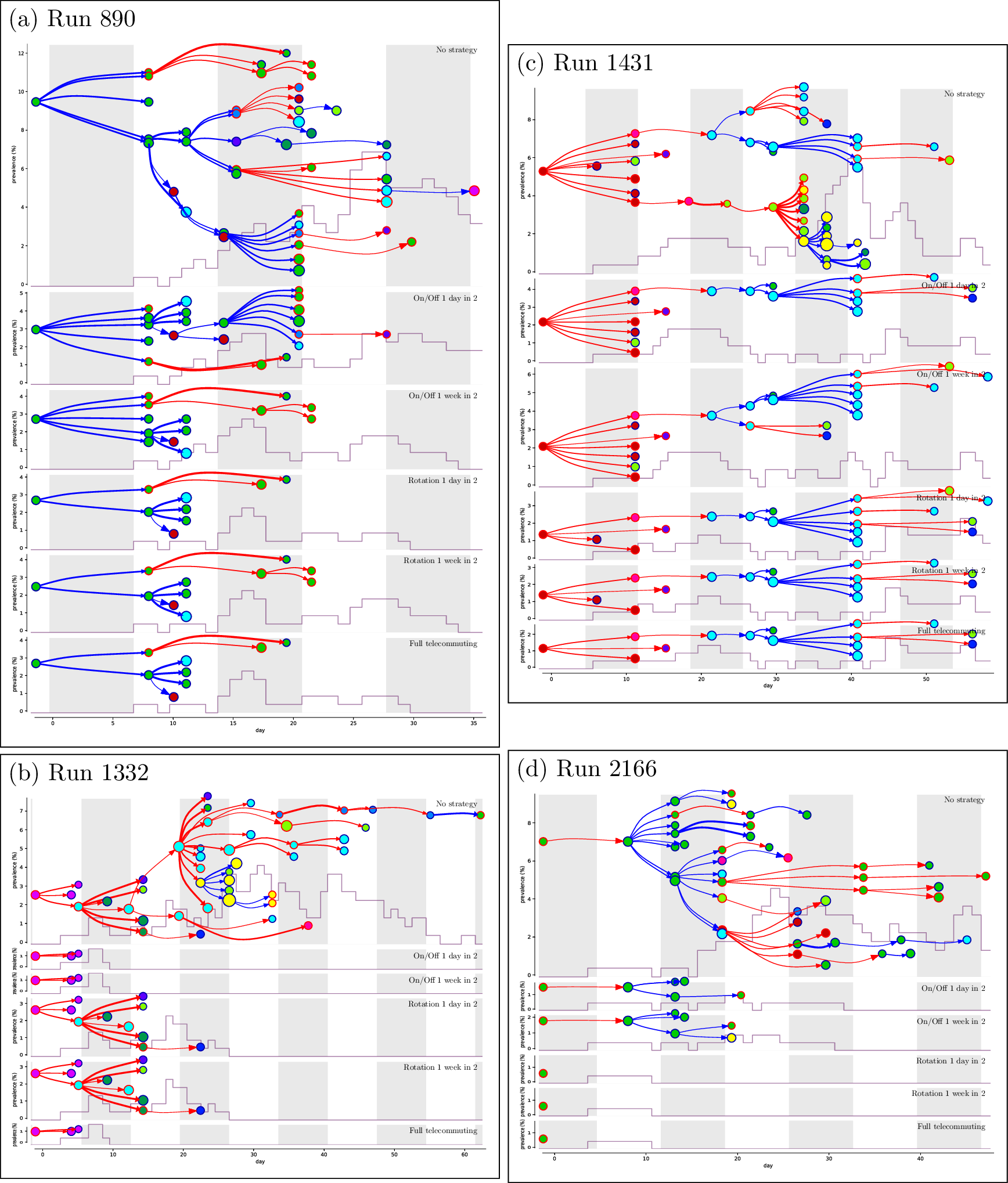

Supplement: S20 Fig — Among the runs producing an outbreak under no strategy, we selected the first four that produce a median number of infections, that is 43. (TIF) [file pcbi.1009264.s021.tif]

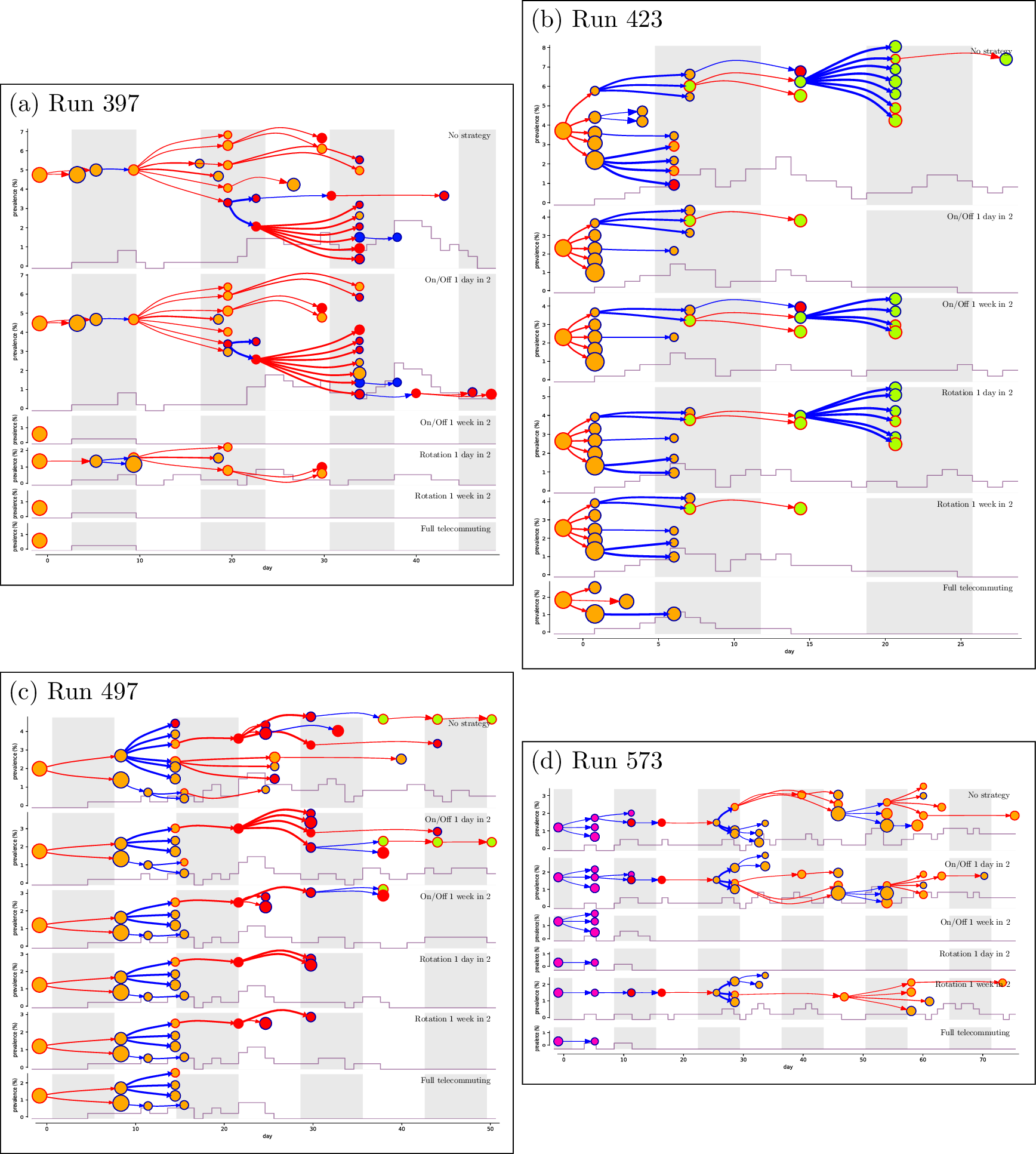

Supplement: S21 Fig — Among the runs producing an outbreak under no strategy, we selected the first four that produce a median number of infections, that is 26. (TIF) [file pcbi.1009264.s022.tif]

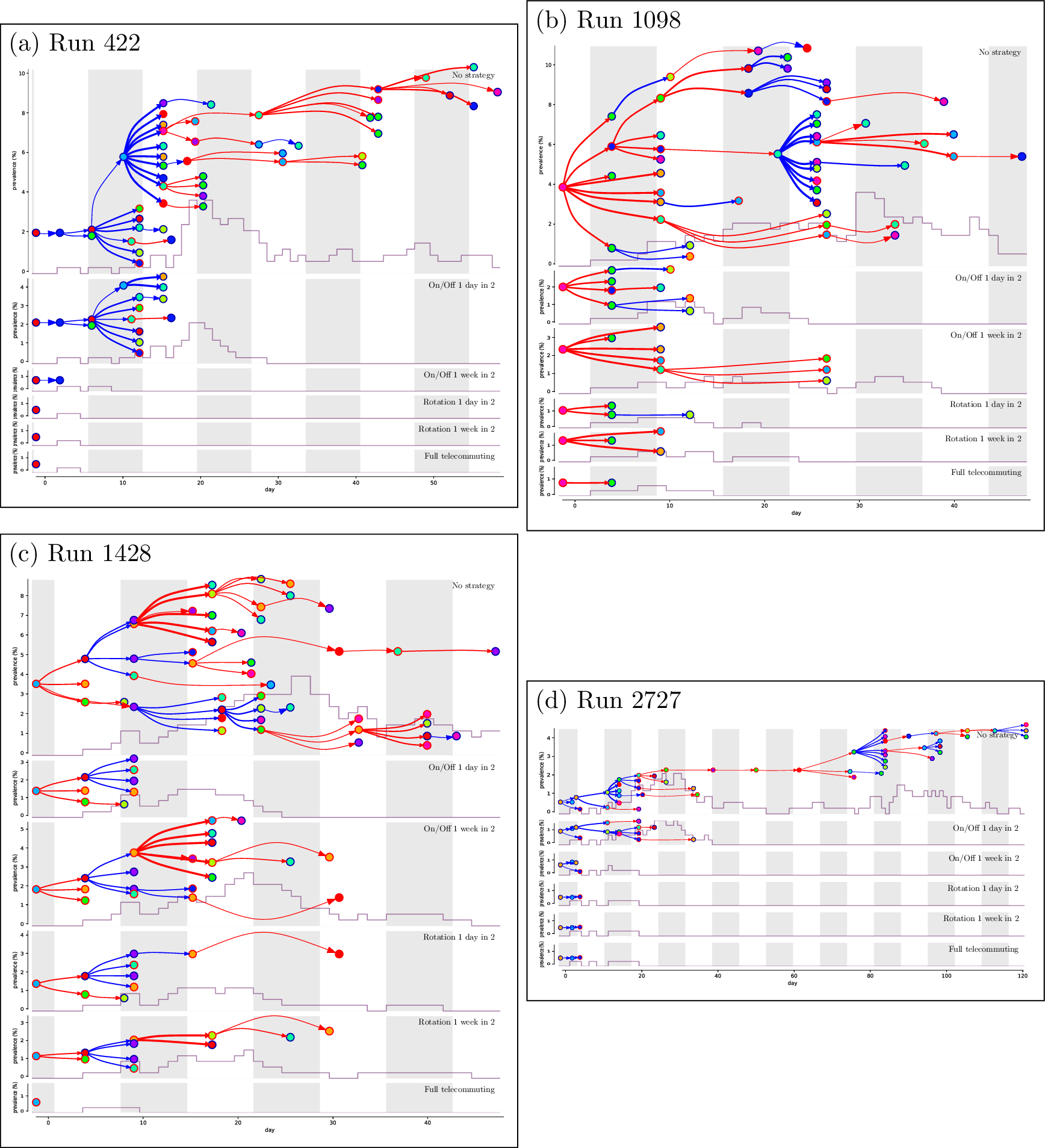

Supplement: S22 Fig — Among the runs producing an outbreak under no strategy, we selected the first four that produce a median number of infections, that is 47. (TIF) [file pcbi.1009264.s023.tif]
