# Supplementary material for: Mitigating COVID-19 outbreaks in workplaces and schools by hybrid telecommuting
Source: PLoS Comput Biol. 2021 Aug 26;17(8):e1009264. doi: 10.1371/journal.pcbi.1009264 (PMC8389398; doi:10.1371/journal.pcbi.1009264)
Supplement: Attachment — Submitted filename: response.pdf [file pcbi.1009264.s024.pdf]

Responses to Reviewers' Questions

Dear editor,

Thank you kindly for the opportunity to revise and resubmit our manuscript.

We would like to thank the two reviewers for their deep reading of our paper and thoughtful feedback. They raised excellent points that we have now addressed in this revised version of the manuscript that we believe is much improved.

Please find below our point-by-point response to all reviewer comments.

We thank you in advance for considering our manuscript and we look forward to hearing from you.

On behalf of all authors, Yours sincerely,

Reviewer #1

The paper simulates the transmission of SARS-CoV-2 in three environments: (i) a primary school, (ii) an office, and (iii) a high school. Results show that Rotating strategies dominate the On-Off strategies which in turns dominates the absence of any policy and that in the long run weekly alternation is a bit better than daily alternation, both for On-Off and for Rotating strategies.

The main limitation of the study that does not seem to be acknowledged in the discussion is the lack of age structure in the model. It is established that the epidemic affects differently people of different ages, for example when it comes to the probability of developing asymptomatic infections, (Davies NG, Klepac P, Liu Y, Prem K, Jit M, Eggo RM. Age-dependent effects in the transmission and control of COVID-19 epidemics. Nature medicine. 2020 Aug;26(8):1205-11.) As different age groups are analysed in these networks, these differences should be considered.

— Response: The reviewer is correct that there is an age-dependent probability of developing asymptomatic vs. symptomatic infections, whereas our simulations assume that 40 per cent of infections are asymptomatic. This is a limitation of our study. In the revised paper, in accordance with the reference, we included a new sensitivity analysis to assess the impact of varying the probability of being asymptomatic for all graphs. This hypothesis did not affect our main conclusions (see Figure S16). We also included a new paragraph to discuss that limit in the discussion section.

A few additional comments:

First line of the abstract, edit the word 'epidemics' with 'epidemic'.

— Response: Done

Lines 32-36 introduce a discussion point about the methodology of the work in a larger context and should be moved in the Discussion paragraph.

— Response: Following the reviewer's comment, we replaced that paragraph by a short sentence "In contrast to most previous work, we focus on real-life and not on synthetically-generated contact networks." and moved the details and references to the Discussion paragraph.

The caption of Fig.2 reports several epidemiological information about the duration of exposed and infectious periods and symptoms, these are inputs to

the model and should all be referenced in the caption as well.

— Response: We thank the reviewer for raising that point which made us realize that it would be more comfortable for the reader if the parameter table was part of the main text. We therefore : (1) added into the legend the following sentence: "We assume that 40% of infected people are asymptomatic." as well as a bibliographical reference [20]; (2) we added references in the caption after each parameter value; and we moved formerly Table S1 to the main text, in the Material and Method section p.10 right after the SuperSpreaders paragraph.

Line 44, change "dissemination of the disease" with "spread of the infection".

— Response: Done

In Methods, subsection contact networks, it should be explained how the authors took into account the missing coverage in the three datasets (4% children, 30% employees, 14% students) as this means that in reality individuals got in contact with a larger number of people, affecting the R0 in the study analysis. If this was not taken into account, the authors should mention it in the study limitations.

— Response: We thank the reviewer for raising that important point. Indeed, imperfect coverage of the studied population was not taken into account. This was motivated by the fact that one of the main points of the paper was to work with real-life, unmodified contact networks. However we believe that this is a very important point and we therefore carried out additional analysis to investigate it. The new version of the article now includes a sensitivity analysis in which we artificially decreased the number of participants in each graph to remove a random fraction, and see that the results still hold (See Figure S18). This analysis confirms that, up to a certain threshold, the fact that contacts graphs were not perfectly observed should not affect our main conclusions. We introduced a paragraph to discuss that specific point in the limitations section.

Lines 63-72 describe the method and should be incorporated in the Strategy section. Each strategy would benefit to be named and presented in a table and then referred when presenting result.

— Response: we modify that paragraph by calling the strategies On-Off Daily, On-Off Weekly, Rotating Daily, Rotating Weekly, and Full Telecommuting. We do not put those definitions in a table since they are best defined in plain text, but put their names in boldface for easier reference.

Reviewer #2

The paper provides an interesting study for the outbreak of Covid-19 epidemic in three real life small contact networks (two schools and one workplace in France), considering two NPI strategies (rotating, with two different time-lags and on-off).

It is well written, theoretically grounded, and easy to follow. As such I only have a few remarks that the authors may want to address in the revision.

1) The analysis has "local" value. How could be in principle the results be extrapolated population to give more general guidelines to policy makers on whether or not follow the "best" rotating strategy. In other words, looking at the national available infection curves (more than one year now) and the

measures implemented in France (for instance in schools), how adherent are the results of the paper on what happened so far? The "retrospective" value of the paper would considerably enhance the analysis.

— Response: Great question. In most places, a variety of measures were implemented concomitantly, making comparison difficult, especially since there was no local information. In France, in May-June 2020, Rotation (or rather, since most schools split the students not at random but tried to preserve existing contacts, something more similar to a version of On-Off) was implemented. No explosion was observed in schools at the time, in accordance with our results. However, no test was available at that time + community prevalence was very low as the population was just out of a general lockdown. With forthcoming generalized testing campaigns in schools and generalized use of autotests, we should be able to have more information about circulation in schools in the near future. We added a discussion section about that specific point in the discussion section.

2) In the current situation there are two main facts impacting on the evolution of the pandemic: (i) the presence of variants (especially the "English" one) , that increase transmissibility (and hence the basic R0) from R0=2.5 to about R0=3.3 (with no restrictions). The computations done in the paper seem not to considere this, if I well understood, (ii) the presence of vaccination - even if in France is still not so massive: is it possible to incorporate in the contact network a vaccination parameters that decreases the susceptibles linearly in time?

— Response: We thank the reviewer for raising that important point. Indeed, the presented work in the paper was done and submitted long before the appearance of variants of concern and the start of vaccinations. However, we agree that in the current context, it is key to evaluate to which extent the analysis is robust with respect to changes in R0 due to circulating variants, or acquired naturally- or vaccination-induced immunity. To address that point we carried out new simulations to explore strategies in situations where: (1) a portion of the population is immune / vaccinated but assessing different characteristics of the immunity conferred by the vaccines (eg partial efficacy at limiting acquisition or transmission) (2) new mutations, conferring higher transmission characteristics to the virus, were selected, in a context where a portion of the population is already immune. Interestingly, the classification of strategies is not changed, although the risk of outbreak obviously varies across all these scenarios. Following this comment, we included that new analysis in the main text, and added two new paragraphs: one at the end of the Material and Methods section and one at the end of the Results section, accompanied by a new Figure (Figure 6) that illustrates these new results.

3) It is clear (and can be proven mathematically) that an on-off strategy is always worst than an off-on strategy, at parity of social-economic costs (that roughly depends only on the closure time interval). The initial amount of infected people is really important and and openings should be suggested only when this number is sufficiently low. I don't see this reasoning in the paper.

— Response: Of course, for epidemic control, the earlier one starts the better, so Off-On is better than On-Off. However, since our strategies are periodic

of period 2 weeks, our simulation assumes that there is a single index patient 0 who is contaminated on a random day between 1 and 14, so there is symmetry and On-Off has the same distribution as Off-On. We added a sentence in the Results section.

4) Could you please give some more details on the stochastic simulations (Gillespy?) and on the underlying stochastic process chain? For individuals (or groups) interactions normally a Markov chain is defined. The overall master model is in general not reducible (marginalizable) at the first moment to get standard epidemiologic detreministic models (like SEIR). Is this the reason of the discrepancy quoted in thye method section?

— Response: We agree that a more technical description of the model implementation was missing. We added a paragraph "Stochastic simulation engine" in the "Material and methods" Section.

We use an agent based model with discrete time where the time step corresponds to a day. Each person is an agent whose state is either S, E, I, or R; according to the SEIR transmission model. Every day, the state of each agent can change according to the number of contacts with other agents in the network for that day, the states of these agents, and random coin flips depending on the transmission probability. This cannot be described easily as a Markov Chain as the state of an agent depends from when it had a previous state change and what random value was obtained for the duration of the current state. Two paragraphs in the "Technical details about the simulation" Section at beginning of the supplementary material further already describe how random values are sampled and how several runs are performed for a given set of parameters.

You are correct that one cannot reduce the evolution of such a model to something solely determined by the average values of the parameters defining it, and that it is the reason of the discrepancy quoted in the method section.

5) In Fig. 3 (medium) the difference in days between no-strategy and full telecommuting is quite low. Are there any reason?

— Response: The reviewer is right, the difference between the two scenarios is low for that specific criterion. This is due to the definition of the criteria: we defined the delay as the time between the virus acquisition of index case and the observation of a cumulative incidence of at least 5 people infected in total, index case not included. The time until that happens, conditioned on the fact that it does happen, is then largely determined by the parameters of the SEIR model, rather than by the choice of strategy. Indeed, considering a single index case, and an average reproductive number of 1.25, at least 2 generations of transmissions are required to reach a cumulative incidence of 5. Considering a generation time between 5 (if symptomatic) and 8 days (if asymptomatic), this would represent between 10 and 15 days. When the effective reproductive number is reduced, this delay is increased. However, it is important to note that the delay is conditioned by the occurrence of an outbreak, whose probability is much lower for the different strategies. We introduced a new paragraph in the Discussion section to discuss that interesting aspect.

6) In Fig. 4, the variable p appears, not defined previously.

— Response: we removed the reference to (not yet defined) p in the caption

of Figure 4.
